# Outcomes, Treatment Patterns, and Resource Use in European Adults with Immune Thrombocytopenia Treated with Avatrombopag

**DOI:** 10.1055/a-2886-7426

**Published:** 2026-07-24

**Authors:** Quentin A. Hill, Francesco Zaja, María Del Carmen Fernandez Sánchez de Mora, Libor Cervinek, Ann Janssens, Fatemeh Saberi Hosnijeh, Luca Le Treust, Fiona Glen, Milica Putnik, Carly Rich, Wolfgang Miesbach

**Affiliations:** 1 Department of Hematology 4472 Leeds Teaching Hospitals NHS TrustLeedsEnglandUnited Kingdom of Great Britain and Northern Ireland; 2 9315 Department of Medical Sciences, University of TriesteTriesteFriuli-Venezia GiuliaItaly; 3 Hematology Unit (SC/UCO) 9247 Azienda Sanitaria Universitaria Giuliano IsontinaTriesteFriuli-Venezia GiuliaItaly; 4 Department of Hematology 16501 Hospital Universitario Reina SofíaCórdobaSpain; 5 Department of Internal Medicine, Hematology and Oncology 532234 University Hospital BrnoBrnoSouth Moravian RegionCzech Republic; 6 Department of Hematology 60182 UZ LeuvenLeuvenFlandersBelgium; 7 Real-World Evidence, Evidence & Access, OPEN HealthRotterdamNetherlands; 8 Real-World Evidence, Evidence & Access, OPEN HealthLondonEnglandUnited Kingdom of Great Britain and Northern Ireland; 9 56772 Swedish Orphan Biovitrum AB PublStockholmStockholm CountySweden; 10 Department of Hemostaseology 14984 Hemophilia Center, University Hospital FrankfurtFrankfurtHEGermany

**Keywords:** clinical studies, platelet immunology, thrombocytopenia, immune thrombocytopenia, thrombopoietin-receptor agonist, platelet count, avatrombopag

## Abstract

**Objective** Clinical trials indicate that avatrombopag, a thrombopoietin-receptor agonist (TPO-RA), is safe and effective for treating patients with primary immune thrombocytopenia (ITP), but real-world evidence on its broader clinical effectiveness is limited.

**Methods** This European multi-center retrospective chart review included adults with primary ITP treated with avatrombopag in routine clinical practice, who had medical records available for ≥ 12 weeks before and after avatrombopag initiation.

**Results** Data from 76 patients with ITP (51 TPO-RA naïve, 25 prior TPO-RA exposed) showed a median time of 4.2 years (interquartile range: 0.7–10.3) from ITP diagnosis to avatrombopag initiation (index date) and a median follow-up of 15.9 months (interquartile range: 7.8–22.8). By week 12, 96 and 93% of the cohort achieved platelet counts (PC) of ≥30 × 10 ^9^ /L and ≥50 × 10 ^9^ /L, respectively; 72% had a complete response (≥100 × 10 ^9^ /L) by week 12, 80% by week 26, and 87% by week 52 postindex. PC reached ≥30 × 10 ^9^ /L, ≥50 × 10 ^9^ /L, and ≥100 × 10 ^9^ /L in a median of 8 (95% confidence interval: 7–12), 10 (8–14), and 19 (12–23) days from the index date, respectively. At 1 year, 75% ( *n* total = 34) and 47% ( *n* total = 21) maintained PC of ≥50 × 10 ^9^ /L and ≥100 × 10 ^9^ /L, respectively. Avatrombopag was permanently discontinued by 19 patients (25%), including 7 (37%) due to being candidates for sustained remission off-treatment. Thromboembolic rates were 4.9 (95% confidence interval: 1.3–12.5) per 100 patient-years during follow-up. The incidence rate (95% confidence interval) of ITP-related outpatient visits and inpatient admissions was 8.9 (8.2–9.5) and 0.1 (0.1–0.2) per person-year.

**Conclusion** This study supports clinical trials’ findings and other postmarketing evidence demonstrating the effectiveness of avatrombopag.

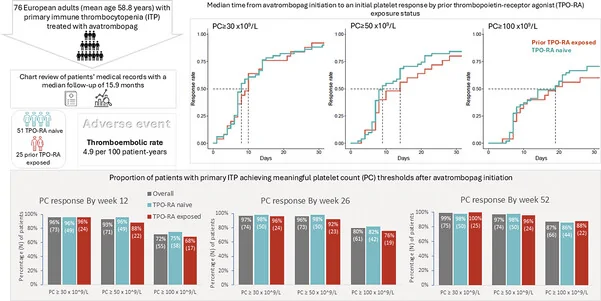

## Introduction

Primary immune thrombocytopenia (ITP) is an autoimmune disorder characterized by isolated thrombocytopenia (peripheral blood platelet count [PC] < 100 × 10 ^9^ /L) in the absence of other causes or disorders that may be associated with thrombocytopenia, and increased bleeding risk. 1 2

Currently available first-line treatments for ITP include agents that decrease platelet destruction (e.g., corticosteroids, intravenous gamma globulin [IVIg]). Second-line treatments include splenectomy, intravenous anti-RhD immunoglobulin, agents that suppress the production of antiplatelet antibodies (e.g., rituximab), 3 4 spleen tyrosine kinase (SYK) inhibitors (e.g., fostamatinib), 5 6 which block Fc-receptor–mediated platelet phagocytosis, and thrombopoietin receptor agonists (TPO-RAs) that stimulate platelet production. Corticosteroids are the standard initial treatment for ITP. They are effective in approximately 70–80% of patients, 7 but side effects limit long-term use, and only 20–40% sustain a durable remission after discontinuation. 8 9 The most common second-line treatments are TPO-RAs (e.g., eltrombopag, romiplostim, avatrombopag), which mimic the biologic effects of endogenous TPO to increase megakaryocyte maturation and platelet production. 10 11 12

Avatrombopag (Doptelet, Sobi) was the most recently approved TPO-RA by the Food and Drug Administration (FDA) in June 2019 and by the European Medicines Agency (EMA) in January 2021, for the treatment of adults with chronic ITP. 3 10 Clinical trials in patients with ITP showed that avatrombopag rapidly increases platelet levels and that this increase was maintained in the longer term for most patients. 13 14 15 However, there are limited real-world studies of avatrombopag treatment in Europe, assessing its clinical effectiveness in a diverse patient population with primary ITP, particularly for patients failing first-line treatment who have not previously received treatment with a TPO-RA. 16 17

This study aimed to describe the treatment patterns and clinical outcomes of European adults with primary ITP treated with avatrombopag, both with and without prior treatment with another TPO-RA. It also provides a comprehensive overview of their characteristics, treatment patterns, and ITP-related healthcare resource utilization (HCRU).

## Methods

### Study Design and Participants

This was a noninterventional, retrospective, multi-center, chart review study based on hospital medical records of patients with primary ITP treated with avatrombopag in 16 centers from six European countries (Belgium, Czech Republic, Germany, Italy, Spain, and the United Kingdom).

Adults (≥18 years) with a confirmed diagnosis of primary ITP, treated with avatrombopag in routine practice, and with ≥12 weeks of medical records before and after avatrombopag initiation were eligible for inclusion. Patients were excluded if they did not provide consent to medical record access, opted out of any research, or participated in interventional studies during follow-up. Participants were recruited between December 13, 2023, and October 30, 2024. Eligible patients were selected consecutively by the direct care team based on avatrombopag treatment initiation date, until center-specific recruitment targets were met. As the study was observational, not hypothesis-driven, and no formal statistical comparisons were planned, the overall sample size was determined primarily by feasibility assessments at each participating site and expert clinical guidance. Recruitment targets for each hospital were set to ensure a balanced contribution across centers. TPO-RA-naïve patients were prioritized, with at least 65% of each center’s recruited patients required to be naïve to prior TPO-RA therapy. This decision was made to align the study population with that of key clinical trials and to allow meaningful contextualization of real-world outcomes. Participants initiated avatrombopag between December 9, 2020, and June 27, 2024. Data were collected from patients’ medical records by the direct care team and transferred to OPEN Health for analysis. Data were fully anonymized, with no personal identifiers collected.

Baseline was defined as the 26 weeks prior to avatrombopag initiation (index date), and the postindex period (follow-up) continued until the latest of last visit, PC assessment, or death. Primary endpoints included the proportion of patients achieving meaningful PC thresholds (≥30 or ≥50 × 10 ^9^ /L) 1 and complete response (≥100 × 10 ^9^ /L) by weeks 12, 26, 52, and at any time while on avatrombopag; time to initial response; proportion with ≥ 50, ≥75%, or doubled increase in PC from baseline among responders; and duration of response. Secondary endpoints included baseline demographics, clinical characteristics, treatment patterns, adverse events of special interest (AESI; including clinically significant bleeding and thromboembolic events [TEs]), and HCRU during follow-up.

Written informed consent was obtained per local regulations. For deceased patients, country-specific consent procedures applied. Research ethics committee, competent authority, and local management approvals for study conduct and pseudonymized data sharing were obtained in line with local regulations.

### Statistical Methods

Analyses were descriptive. Quantitative variables were summarized using mean, standard deviation (SD), medians, and interquartile range (IQR); categorical variables with frequencies and percentages. Point estimates for proportions were presented with associated 95% confidence intervals (CI). Kaplan–Meier methods estimated the time to and duration of response. Treatment duration was evaluated from the date of avatrombopag initiation to the documented date of discontinuation, with right-censoring for ongoing treatment, unrelated deaths, or loss to follow-up.

Baseline values were taken from the closest measurement to the index date within the baseline period, except for PC. Baseline PC was defined as the median of up to three PC observations closest to avatrombopag initiation; if a patient had two PC observations in the baseline period, the mean of those values was used; if only one, that value was used.

Durability was measured from the first response to avatrombopag until the first date the response criteria were no longer met. Loss of response was defined as two or more consecutive PCs below threshold, at least 1 week apart; loss of response date was the first low PC in that series. Response was considered regained when a patient re-achieved a response level PC. Sensitivity analyses were conducted using 2- and 4-week gaps between consecutive PC observations to assess the robustness of the primary definition. PC values obtained during rescue therapy were excluded from the analysis. For analyses describing an event occurring by a particular time point (e.g., by week 12), all measurements between the index date and that time point were included while for analyses considering an event occurring at a specific time period (e.g., at baseline, at week 12) an appropriate window (e.g., ±4 weeks) was applied around this time point to account for variability in appointment scheduling in real-world practice.

For each AESI, incidence rates per 100 patient-years were calculated by dividing the total number of specific AE by the total patient-years at risk (from index to discontinuation or end of follow-up), then multiplying by 100. Additionally, a time-to-event analysis was conducted for each AESI type using the Mean Cumulative Function model, 18 with death or avatrombopag discontinuation as competing or terminal events, and censoring at the end of follow-up.

For annualized HCRU (number of visits/admissions, length of stay), incidence rates (IR; e.g., number of outpatient visits per patient-year) and 95% CI were reported using the Exact method. 19 Missing data were not imputed, but the number of missing values was reported. Statistical analyses were conducted using R statistical software (version 4.4.2).

## Results

### Patients’ Characteristics

Data were collected for 76 patients (6 [8%] from Belgium, 11 [14%] from Czech Republic, 9 [12%] from Germany, 10 [13%] from Italy, 20 [26%] from Spain, and 20 [26%] from United Kingdom), of whom 51 (67%) were TPO-RA naïve and 25 (33%) were TPO-RA exposed ( Table 1 ). The mean (SD) age at ITP diagnosis and avatrombopag initiation was 50.8 (21.3) and 58.8 (19.1) years, respectively. Around half (54%, *n* = 41) of patients were female. Sex, age at avatrombopag initiation, and ethnicity distributions were similar across the TPO-RA naïve and TPO-RA exposed groups.

**Table 1:** Baseline demographics, clinical characteristics, and treatment history of patients with primary ITP.

	Overall, *n* = 76	TPO-RA naïve, *n* = 51	TPO-RA exposed, *n* = 25
Age at data collection, years, mean (SD)	58.8 (19.1)	59.5 (19.3)	57.3 (18.9)
Age at ITP diagnosis, years, mean (SD)	50.8 (21.3)	54.9 (20.0)	42.1 (21.8)
Female sex, *n* (%)	41 (54%)	27 (53%)	14 (56%)
Ethnicity, *n* (%)			
European	66 (87%)	43 (84%)	23 (92%)
Hispanic	4 (5%)	4 (8%)	0
Middle Eastern	1 (1%)	0	1 (4%)
Not stated	5 (7%)	4 (8%)	1 (4%)
Country, *n* (%)			
Belgium	6 (8%)	6 (12%)	0
Czech Republic	11 (14%)	9 (18%)	2 (8%)
Germany	9 (12%)	4 (8%)	5 (20%)
Italy	10 (13%)	9 (18%)	1 (4%)
Spain	20 (26%)	14 (27%)	6 (24%)
United Kingdom	20 (26%)	9 (18%)	11 (44%)
Time from diagnosis to index, years, mean (SD)	7.9 (10.5)	4.8 (5.8)	14.3 (14.7)
Median (IQR)	4.2 (0.7–10.3)	3.0 (0.3–6.7)	8.7 (3.9–20.0)
≤3 mo from diagnosis to index, *n* (%)	13 (17%)	12 (24%)	1 (4%)
3–12 mo from diagnosis to index, *n* (%)	6 (8%)	5 (10%)	1 (4%)
>12 mo from diagnosis to index, *n* (%)	55 (72%)	33 (65%)	22 (88%)
Not available	2 (3%)	1 (2%)	1 (4%)
Follow-up, months, mean (SD)	15.8 (9.0)	15.4 (8.0)	16.8 (11.0)
Median (IQR)	15.9 (7.8–22.8)	16.4 (8.0–22.1)	13.3 (7.5–26.2)
Baseline platelet count, ×10 ^9^ /L, Median (IQR) ^a^	42.0 (23.2–87.5)	37.0 (24.0–85.0)	46.0 (16.0–88.0)
Bone marrow biopsy to confirm ITP, *n* (%)	20 (26%)	14 (27%)	6 (24%)
Missing	10 (13%)	3 (6%)	7 (28%)
History of splenectomy, *n* (%)	5 (7%)	0	5 (20%)
Missing	5 (7%)	4 (8%)	1 (4%)
Prior steroid medications, *n* (%)	57 (75%)	41 (80%)	16 (64%)
Dexamethasone	19 (25%)	10 (20%)	9 (36%)
Prednisolone/prednisone	47 (62%)	34 (66%)	13 (52%)
Other ^b^	4 (5%)	4 (8%)	0
Prior nonsteroidal medications, *n* (%)	30 (39%)	15 (29%)	15 (60%)
IVIg	20 (26%)	10 (20%)	10 (40%)
Azathioprine	6 (8%)	2 (4%)	4 (16%)
Mycophenolate mofetil	5 (7%)	2 (4%)	3 (12%)
Rituximab	4 (5%)	2 (4%)	2 (8%)
Cyclosporine	2 (3%)	2 (4%)	0
Dapsone	2 (3%)	2 (4%)	0
Fostamatinib disodium	2 (3%)	0	2 (8%)
Other ^c^	2 (3%)	0	2 (8%)
Prior TPO-RA medication, *n* (%)	25 (33%)	NR	25 (100%)
Romiplostim only	3 (4%)	NR	3 (12%)
Eltrombopag only	16 (21%)	NR	16 (64%)
Romiplostim and eltrombopag	6 (8%)	NR	6 (24%)
Duration of eltrombopag treatment, days, Median (IQR)	308.5 (64.2–553.5)	NR	308.5 (64.2–553.5)
Duration of romiplostim treatment, days, median (IQR)	475.0 (161.0–612.0)	NR	475.0 (161.0–612.0)
Reasons for switching from prior TPO-RA to avatrombopag, *n* (%) ^d^			
Lack of response	7 (9%)	NR	7 (28%)
Convenience	6 (8%)	NR	6 (24%)
Relapse	4 (5%)	NR	4 (16%)
Intolerance	2 (3%)	NR	2 (8%)
Other ^e^	4 (5%)	NR	4 (16%)
Missing	2 (3%)	NR	2 (8%)

Abbreviations: IQR, interquartile range; ITP, immune thrombocytopenia; NR, not relevant; SD, standard deviation; TPO-RA, thrombopoietin-receptor agonist.

Note: Data on steroid/nonsteroid medications included patients with a prescription of steroid/nonsteroid started prior to the index date, but could be stopped or ongoing at the index.

^a^ Six patients with missing baseline platelet count (PC). Distribution of the baseline PC were as follow: PC < 30 × 10 ^9^ /L: 24/70 (34.3%), PC ≥ 30 × 10 ^9^ /L to <50 × 10 ^9^ /L: 17/70 (24.3%), PC ≥ 50 × 10 ^9^ /L to <100 × 10 ^9^ /L: 15/70 (21.4%), PC ≥ 100 × 10 ^9^ /L: 14/70 (20.0%).

^b^ For overall and TPO-RA-naïve cohort: methylprednisolone: *n* = 4.

^c^ For overall and TPO-RA exposed cohort: danazol ( *n* = 1), anti-RhD immunoglobulin ( *n* = 1).

^d^ The reason for switching from the most recent TPO-RA per type of medication: for eltrombopag ( *n* = 18): lack of response *n* = 5, convenience *n* = 3, relapse *n* = 4, intolerance *n* = 2, others *n* = 3, missing *n* = 1; for romiplostim ( *n* = 7): lack of response *n* = 2, convenience *n* = 3, others *n* = 1, missing *n* = 1.

^e^ Including deranged liver function test ( *n* = 1), eating disorder ( *n* = 1), fluctuating platelet numbers ( *n* = 2).

The median (IQR) time from ITP diagnosis until avatrombopag initiation was 4.2 (0.7–10.3) years, and patients were followed for a median time of 15.9 (7.8–22.8) months. At index, 17% were newly diagnosed (≤3 months), 8% had persistent (3–12 months), and 72% had chronic ITP (>12 months). Thirty-four percent ( *n* = 17) of TPO-RA-naïve patients were diagnosed ≤12 months before avatrombopag initiation versus 8% ( *n* = 2) in the exposed group. Median (IQR) baseline PC was 42.0 × 10 ^9^ /L (23.2 × 10 ^9^ –87.5 × 10 ^9^ L) in the overall cohort, 46.0 × 10 ^9^ /L (16.0 × 10 ^9^ –88.0 × 10 ^9^ L) in the TPO-RA exposed group, and 37.0 × 10 ^9^ /L (24.0 × 10 ^9^ –85.0 × 10 ^9^ L) in TPO-RA naïve patients.

### Treatment History

Approximately 75% ( *n* = 57) of patients received steroids during baseline ( Table 1 ); most commonly prednisolone/prednisone (62%, *n* = 47), and dexamethasone (25%, *n* = 19). Overall, 39% ( *n* = 30) used at least one nonsteroid or non-TPO-RA treatment during baseline, most commonly IVIg (26%, *n* = 20). Among TPO-RA exposed patients ( *n* = 25), 64% ( *n* = 16) had prior eltrombopag only, 12% ( *n* = 3) romiplostim only, and 24% ( *n* = 6) both during baseline. The main reasons for switching from a prior TPO-RA to avatrombopag were lack of response (28%, *n* = 7), convenience (24%, *n* = 6), and relapse (16%, *n* = 4; Table 1 ).

The majority of patients (89%, *n* = 68) started avatrombopag at 20 mg once daily ( Table 2 ). Approximately 80% had a change in avatrombopag dosage (with 34% requiring a dose increase and 58% a dose decrease) during the follow-up period, which was similar for TPO-RA naïve and exposed groups. The median (IQR) duration of avatrombopag treatment was 328.5 days (129.8–671.0) for the overall cohort, and 71.5% (95% CI: 61.1–83.7) of patients continued avatrombopag for at least 2 years (TPO-RA naïve: 74.1% [62.2–88.2], TPO-RA exposed: 65.3% [46.3–92.1]). Twenty-six patients (34%) had ongoing steroid treatment at avatrombopag initiation. Of these, 25 (96%) were able to discontinue ( *n* = 22) or reduce ( *n* = 3) steroid treatment during the follow-up period ( Table 2 ).

**Table 2:** Avatrombopag treatment.

	Overall, *n* = 76	TPO-RA naïve, *n* = 51	TPO-RA exposed, *n* = 25
Dose of avatrombopag at initiation, *n* (%)			
20 mg once daily	68 (89%)	47 (92%)	21 (84%)
40 mg once daily	3 (4%)	0	3 (12%)
Other ^a^	5 (7%)	4 (8%)	1 (4%)
Time on avatrombopag treatment postindex, days, Median (IQR)	328.5 (129.8–671.0)	335.0 (156.0–662.5)	323.0 (121.0–687.0)
Any change in dose of avatrombopag postindex date, *n* (%)	59 (78%)	40 (78%)	19 (76%)
Dose increase ^b^	26 (34%)	17 (33%)	9 (36%)
Dose decrease ^b^	44 (58%)	32 (63%)	12 (48%)
Avatrombopag permanent discontinuation postindex, *n* (%)	19 (25%)	12 (24%)	7 (28%)
Reason for avatrombopag discontinuation, *n* (% from patients who discontinued avatrombopag)			
Having a maintained response/being deemed a candidate for achieving sustained remission off-treatment ^c^	7 (37%)	5 (42%)	2 (29%)
Intolerance	5 (26%)	3 (25%)	2 (29%)
Lack of response	3 (16%)	3 (25%)	0
Person’s choice	2 (11%)	0	2 (29%)
Other ^d^	2 (11%)	1 (8%)	1 (14%)
Proportion of patients remaining on avatrombopag at time point			
1 y (95% CI)	73.7% (63.7–85.3%)	77.1% (66.0–90.2%)	65.3% (46.3–92.1%)
2 y (95% CI)	71.5% (61.1–83.7%)	74.1% (62.2–88.2%)	65.3% (46.3–92.1%)
3 y (95% CI)	71.5% (61.1–83.7%)	NE	65.3% (46.3–92.1%)
Ongoing steroid treatment at index, *n* (%) ^e^	26 (34%)	21 (41%)	5 (20%)
Steroid dose reduction postindex, *n* (%)	15 (58%)	11 (52%)	4 (80%)
Steroid discontinuation postindex, *n* (%)	22 (85%)	20 (95%)	2 (40%)
Concomitant ITP treatments ^f^			
Nonsteroids, *n* (%)	16 (21%)	8 (16%)	8 (32%)
Steroids, *n* (%)	36 (47%)	29 (57%)	7 (28%)
Patients with rescue medication prescribed postindex while on avatrombopag, *n* (%)	12 (16%)	6 (12%)	6 (24%)
Time from index to first rescue treatment, days, Median (IQR)	91 (28–141)	102.5 (63.8–183)	52 (25–100)
New ITP treatment after avatrombopag discontinuation postindex, *n*	10 out of 19	6 out of 12	4 out of 7
Eltrombopag	5	3	2
Romiplostim	5	3	2

Abbreviations: CI, confidence intervals; IQR, interquartile range; ITP, immune thrombocytopenia; NE, not estimable; TPO-RA, thrombopoietin-receptor agonist.

^a^ TPO-RA naïve: 20 mg every other day ( *n* = 1), 20 mg three times a week ( *n* = 1), 20 mg four times a week ( *n* = 1), 40 mg three times a week and 20 mg on the four remaining days of each week ( *n* = 1); TPO-RA exposed: 40 mg three times a week and 20 mg on the four remaining days of each week ( *n* = 1).

^b^ Any increase or decrease from the initial dose; numbers are not mutually exclusive.

^c^ Of those who discontinued due to having a maintained response and/or being a candidate for SROT, all seven achieved complete response before treatment discontinuation. The median (IQR) time from initiation of avatrombopag to initiation of tapering and to discontinuation, respectively, was 20 days (16–25) and 297 days (60–333). Between discontinuing avatrombopag and the time of last follow-up, no patient had a relapse requiring treatment, with a median (IQR) of 366 days (266–542).

^d^ Including adverse event ( *n* = 1, TPO-RA naïve), development of edema ( *n* = 1, TPO-RA exposed).

^e^ Numbers are not mutually exclusive; 12 patients had a steroid dosage reduction and later discontinued the steroid treatment.

^f^ Concomitant medications: both medications that were ongoing at index and those newly initiated postindex.

Avatrombopag treatment was permanently discontinued postindex by 25% ( *n* = 19: 12 TPO-RA naïve and 7 TPO-RA exposed) of patients, most commonly due to having a maintained response/being deemed a candidate for achieving sustained remission off-treatment (SROT; 37%, *n* = 7), intolerance (26%, *n* = 5), and lack of response (16%, *n* = 3; Table 2 ).

In total, 12 patients (16%) were prescribed rescue medications while they were on avatrombopag postindex (TPO-RA naïve: 6 [12%], TPO-RA exposed: 6 [24%]). The median (IQR) time from avatrombopag initiation to first rescue treatment was 91.0 days (28.0–141.0) in the overall cohort, 102.5 days (63.8–183.0) in the TPO-RA-naïve, and 52.0 days (25.0–100.0) in the TPO-RA-exposed group ( Table 2 ).

### Proportion of Patients Achieving Meaningful PC Thresholds Postindex

**Fig. 1:**
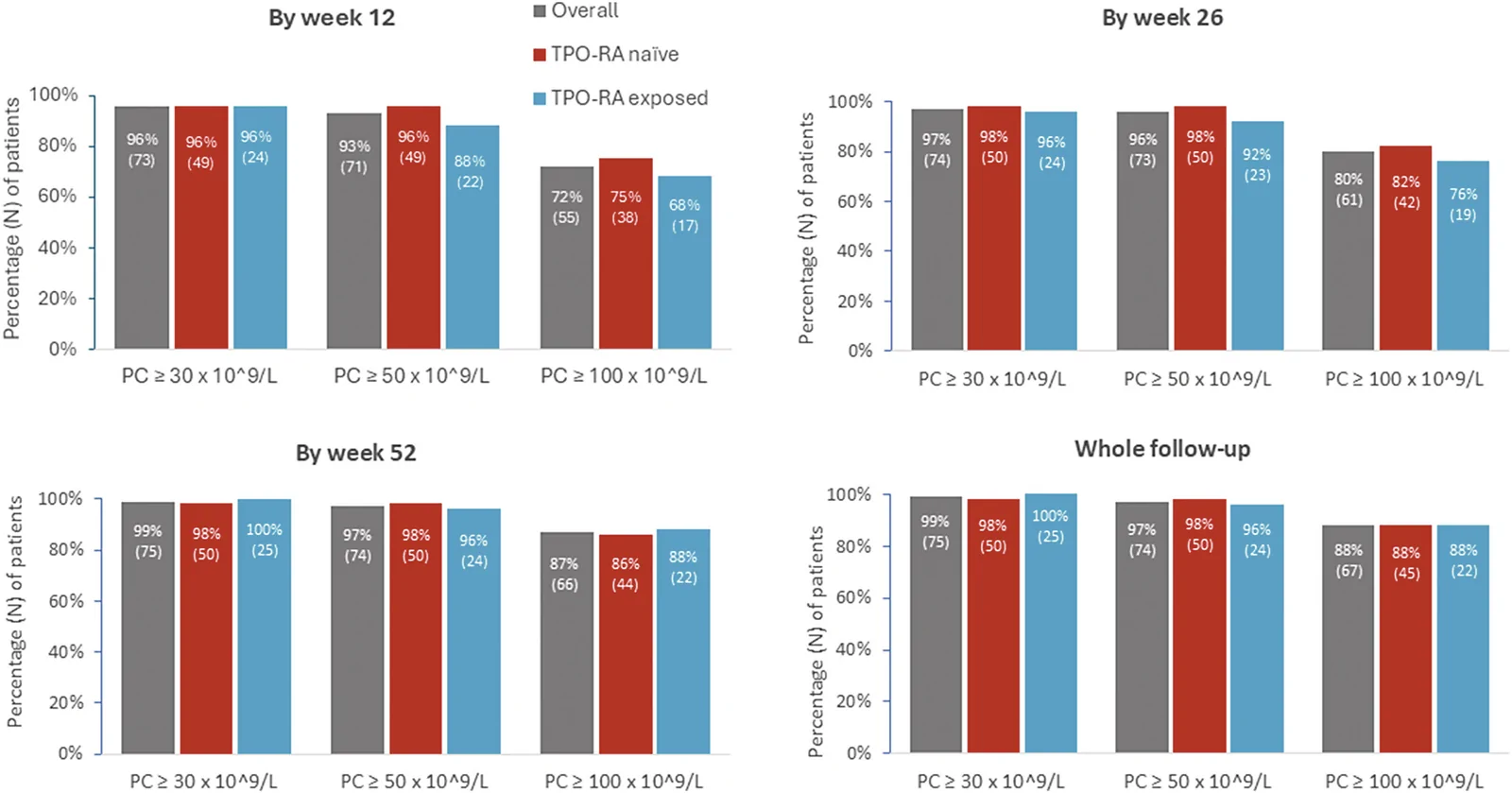
Proportion of patients with primary ITP achieving meaningful PC thresholds postindex by predefined time points, overall and by prior TPO-RA exposure status. For analyses describing the proportion of patients who achieved a PC threshold by a particular time point (e.g., by week 12), all measurements between the index date and that time point were included. Therefore, the reported percentages indicate cumulative responders, not sustained responders. The total cohort size ( *n* = 76) was used as the denominator to provide a conservative estimate of response. Due to variability in follow-up duration, the reported percentages reflect minimum response rates. ITP, immune thrombocytopenia; PC, platelet count; TPO-RA, thrombopoietin-receptor agonist.

By week 12, 96% ( *n* = 73) and 93% ( *n* = 71) of the patients cumulatively achieved PC threshold of 30 × 10 ^9^ /L and 50 × 10 ^9^ /L, respectively, at any time up to that point ( Fig. 1 ). Seventy-two percent (55/76) had at least once complete response (≥100 × 10 ^9^ /L) by week 12, 80% (61/76) by week 26, and 87% (66/76) by week 52 postindex. Eighty-eight percent (67/76) had a record of complete response at any time postindex. Subgroup analysis of chronic ITP patients (diagnosed > 12 months ago; *n* = 55) showed findings consistent with the overall cohort ( **Supplementary Fig. S1** ). The sample sizes of the newly diagnosed ( *n* = 13) and persistent ( *n* = 6) subgroups were insufficient to permit robust statistical analysis.

PC doubled from baseline in 70% (53/76), 72% (55/76), and 75% (57/76) of patients by weeks 12, 26, and 52, respectively ( **Supplementary Fig. S2** ). In general, the proportion of patients with a ≥100% increase in PC from baseline by weeks 12, 26, and 52 was numerically higher in the TPO-RA exposed (72%/76%/80%) compared with the TPO-RA naïve group (69%/71%/73%).

**Supplementary Fig. S3** shows the proportion of patients achieving meaningful PC thresholds at each specified time point. Overall, 45% (34/76) of patients had a complete response (≥100 × 10 ^9^ /L) at week 12, 37% (28/76) at week 26, and 22% (17/76) at week 52 postindex. While Fig. 1 indicates that a high proportion of patients achieved a clinically meaningful PC response at least once during treatment, **Supplementary Fig. S3** presents PC results at predefined time points. Across these time points, the proportion of patients meeting established response thresholds varied over time, reflecting variability in PC responses in real-world ITP practice. The median (IQR) percentage change in PC from baseline was 160% (45–380) at week 12, 193% (34–525) at week 26, and 144% (38–397) at week 52 ( **Supplementary Table S1** ).

### Time from Index Date to an Initial Platelet Response

**Fig. 2:**
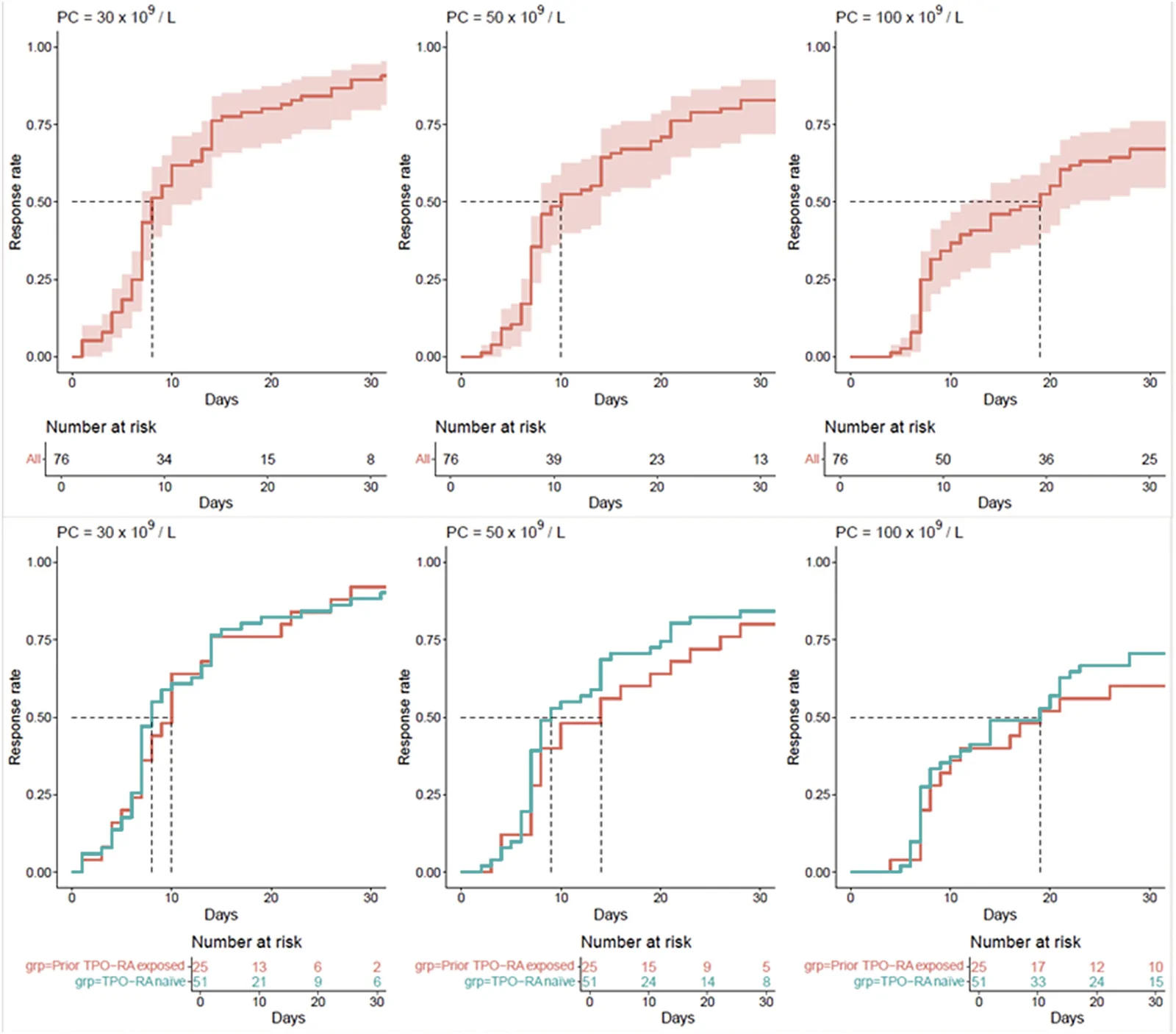
Median time from index date to an initial platelet response, overall (upper row) and by prior TPO-RA exposure status (lower row). The “number at risk” refers to the total number of patients who are still being observed and have not yet experienced the event of interest (PC response) at the beginning of each time period. This number decreases over time as events occur or as patients are censored. The red-shaded area in the top row represents the 95% confidence intervals. The blue line in the lower row indicates the TPO-RA naïve group, while the red line represents the TPO-RA exposed group. PC, platelet count; TPO-RA, thrombopoietin-receptor agonist.

The median (95% CI) time to achieve a PC of ≥30 × 10 ^9^ /L, ≥50 × 10 ^9^ /L, and ≥100 × 10 ^9^ /L was 8 (7–12) days, 10 (8–14) days, and 19 (12–23) days, respectively, from the index date ( Fig. 2 ). The median (95% CI) time to achieve a complete response was similar for TPO-RA naïve (19 days [11–23]) and exposed (19 days [10–154]) patients.

In chronic ITP patients, the median (95% CI) time to achieve a PC of ≥30 × 10 ^9^ /L, ≥50 × 10 ^9^ /L, and ≥100 × 10 ^9^ /L was 9 (7–13) days, 10 (8–15) days, and 17 (11–26) days, respectively, which were not meaningfully different from the overall data ( **Supplementary Fig. S4** ). Median time to complete response was 14 (8–23) days for TPO-RA naïve and 23.5 days (16–202) for previously exposed patients.

### Duration of Response

Of patients remaining on avatrombopag who achieved PC of ≥50 × 10 ^9^ /L and ≥100 × 10 ^9^ /L, 75% (95% CI: 65–86, *n* = 34) and 47% (36–62, *n* = 21) maintained the response at 1 year after initial response, respectively ( Table 3 ). Of patients who achieved a PC of ≥50 × 10 ^9^ /L, 70% (58–85, *n* = 11), 81% (71–93, *n* = 7), and 49% (28–86, *n* = 4) maintained the response at 2 years in the overall cohort, TPO-RA naïve, and TPO-RA exposed groups, respectively. Sensitivity analyses using 2- and 4-week gaps between consecutive PC observations yielded results that were consistent with the primary analysis based on a 1-week gap. These analyses confirmed that varying the gap definition did not meaningfully affect the estimated proportions of patients maintaining each PC threshold, demonstrating the robustness of the findings ( **Supplementary Table S2** ).

**Table 3:** Proportion (95% CI) of patients with maintained response at 1 and 2 years (in responders).

Overall cohort					
PC response	Maintained response at		Overall, *n* = 76	TPO-RA naïve, *n* = 51	TPO-RA exposed, *n* = 25
≥30 × 10 ^9^ /L	Year 1	Proportion (95% CI)	90.6% (84.3–97.5%)	92.0% (84.8–99.8%)	88.0% (76.1–100%)
		*n* patients at time 0	75	50	25
		*n* at risk at the start of year 1	42	30	12
		*n* events (between years 0 and 1)	7	4	3
	Year 2	Proportion (95% CI)	90.6% (84.3–97.5%)	92.0% (84.8–99.8%)	88.0% (76.1–100%)
		*n* patients at time 0	75	50	25
		*n* at risk at the start of year 2	15	8	7
		*n* events (between years 1 and 2)	0	0	0
≥50 × 10 ^9^ /L	Year 1	Proportion (95% CI)	75.1% (65.4–86.3%)	81.1% (70.6–93.1%)	61.4% (42.9–87.8%)
		*n* patients at time 0	74	50	24
		*n* at risk at the start of year 1	34	27	7
		*n* events (between years 0 and 1)	17	9	8
	Year 2	Proportion (95% CI)	70.1% (57.8–85.1%)	81.1% (70.6–93.1%)	49.1% (27.9–86.4%)
		*n* patients at time 0	74	50	24
		*n* at risk at the start of year 2	11	7	4
		*n* events (between years 1 and 2)	1	0	1
≥100 × 10 ^9^ /L	Year 1	Proportion (95% CI)	47.1% (35.8–62.0%)	55.7% (42.1–73.7%)	29.6% (14.6–60.1%)
		*n* patients at time 0	67	45	22
		*n* at risk at the start of year 1	21	17	4
		*n* events (between years 0 and 1)	32	18	14
	Year 2	Proportion (95% CI)	43.2% (31.3–59.7%)	55.7% (42.1–73.7%)	NE
		*n* patients at time 0	67	45	22
		*n* at risk at the start of year 2	3	3	0
		*n* events (between years 1 and 2)	1	0	0
Patients with chronic ITP (diagnosed > 12 mo prior to treatment initiation)			*n* = 55	*n* = 33	*n* = 22
≥30 × 10 ^9^ /L	Year 1	Proportion (95% CI)	90.7% (83.3–98.8%)	93.8% (85.7–100%)	86.4% (73.2–100%)
		*n* patients at time 0	54	32	22
		*n* at risk at the start of year 1	31	21	10
		*n* events (between years 0 and 1)	5	2	3
	Year 2	Proportion (95% CI)	90.7% (83.3–98.8%)	93.8% (85.7–100%)	86.4% (73.2–100%)
		*n* patients at time 0	54	32	22
		*n* at risk at the start of year 2	12	6	6
		*n* events (between years 1 and 2)	0	0	0
≥50 × 10 ^9^ /L	Year 1	Proportion (95% CI)	75.1% (63.6–88.7%)	86.8% (75.6–99.8%)	55.6% (35.9–85.9%)
		*n* patients at time 0	53	32	21
		*n* at risk at the start of year 1	24	19	5
		*n* events (between years 0 and 1)	12	4	8
	Year 2	Proportion (95% CI)	68.8% (54.3–87.3%)	86.8% (75.6–99.8%)	41.7% (20.4–85.1%)
		*n* patients at time 0	53	32	21
		*n* at risk at the start of year 2	8	5	3
		*n* events (between years 1 and 2)	1	0	1
≥100 × 10 ^9^ /L	Year 1	Proportion (95% CI)	34.4% (22.5–52.7%)	41.9% (26.7–65.9%)	22.8% (8.9–58.7%)
		*n* patients at time 0	49	30	19
		*n* at risk at the start of year 1	12	10	2
		*n* events (between years 0 and 1)	29	16	13
	Year 2	Proportion (95% CI)	34.4% (22.5–52.7%)	41.9% (26.7–65.9%)	NE
		*n* patients at time 0	49	30	19
		*n* at risk at the start of year 2	2	2	0
		*n* events (between years 1 and 2)	0	0	0

Abbreviations: CI, confidence intervals; NE, not estimable; TPO-RA, thrombopoietin-receptor agonist.

Note: Proportion and 95% confidence intervals were calculated using the Kaplan–Meier estimator, based on the number of events and the number at risk at specific time points. Loss of response was defined as two or more consecutive PCs below threshold, at least 1 week apart.

### Adverse Events and Mortality

Nine patients (12%) experienced bleeding events (clinically significant or common terminology criteria for adverse events (CTCAE) grade ≥ 3), and 3 (4%) had TEs while on avatrombopag ( Table 4 ). In total, there were 19 bleeding events and 4 TEs, which included jugular vein thrombosis ( *n* = 1, treatment was continued with the initial dose), pulmonary embolism ( *n* = 2, treatment was discontinued), and right renal vein thrombosis with floating thrombus in the inferior cava ( *n* = 1, treatment was discontinued). During follow-up, bleeding and TE rates were 23.1 (95% CI: 13.9–36.1) and 4.9 (1.3–12.5) per 100 patient-years, respectively. The median (IQR) PC at the bleeding event (based on the closest PC to the event within 0–4 days, *n* = 18 events) was 53.5 (10.2–78.2). The TEs occurred in females aged 47–62 years, with PC ranging from 25 × 10 ^9^ /L to 256 × 10 ^9^ /L. TEs occurred primarily within the first 6 months of treatment, whereas bleeding events were observed throughout the first 2 years ( **Supplementary Fig. S5** ). Two participants died postindex, but their deaths were not related to avatrombopag ( Table 4 ).

**Table 4:** Thromboembolic and bleeding events and mortality postindex.

	Overall, *n* = 76	TPO-RA naïve, *n* = 51	TPO-RA exposed, *n* = 25
Patients experienced AE postindex, *n* (%)	12 (16%)	9 (18%)	3 (12%)
Patients with thromboembolic events, *n* (%) ^a^			
Anytime ^b^	3 (4%)	2 (4%)	1 (4%)
By week 12	2 (3%)	1 (2%)	1 (4%)
By week 26	3 (4%)	2 (4%)	1 (4%)
By week 52	3 (4%)	2 (4%)	1 (4%)
Patients with bleeding events, *n* (%) ^a^			
Anytime	9 (12%)	7 (14%)	2 (8%)
By week 12	5 (7%)	4 (8%)	1 (4%)
By week 26	6 (8%)	5 (10%)	1 (4%)
By week 52	8 (11%)	6 (12%)	2 (8%)
PC at bleeding, mean (SD) ^c^	48.7 (36.0)	51.7 (35.3)	40.8 (40.8)
Median (IQR)	53.5 (10.2–78.2)	59.0 (20.0–79.0)	34.0 (7.0–52.0)
Adverse events, *n*			
Bleeding event—clinically significant	19	14	5
IR (95% CI), per 100 patient-years	23.1 (13.9–36.1)	25.6 (14.0–42.9)	18.2 (5.9–42.5)
Thromboembolic event	4	3	1
Jugular vein thrombosis	1	0	1
Pulmonary embolism	2	2	0
Right renal vein thrombosis with floating thrombus in inferior cava	1	1	0
IR (95% CI), per 100 patient-years	4.9 (1.3–12.5)	5.5 (1.1–16.0)	3.6 (0.1–20.3)
Death postindex, *n* (%)	2 (3%)	2 (4%)	0
Avatrombopag-related death	0	0	0

Abbreviations: AE, adverse events; CI, confidence intervals; IQR, interquartile range; IR, incidence rate; SD, standard deviation; TPO-RA, thrombopoietin-receptor agonist.

^a^ Categories are not mutually exclusive, clinically significant, or Common Terminology Criteria for Adverse Events (CTCAE) Grade 3+ bleeding.

^b^ Platelet count at thromboembolic events included 25 × 10 ^9^ /L ( *n* = 1, on event date), 62 × 10 ^9^ /L ( *n* = 1, 1 day prior to event), and 256 × 10 ^9^ /L ( *n* = 1, 11 days prior to the event).

^c^ Platelet count closest to the date of the bleeding event ( *n* = 18 events: TPO-RA naïve = 13 events, TPO-RA exposed = 5 events) within a few days (0–4 days before or after the date of the bleeding event). Including events with platelet count on the same day as the bleeding event ( *n* = 11 events): overall mean (SD): 33.3 (33.3), overall median (IQR): 20.0 (5.5–61.0), TPO-RA naïve (nine events): mean (SD): 39.2 (34.2), median (IQR): 34.0 (5.0–63.0), TPO-RA exposed (two events): mean (SD): 6.5 (0.7), median (IQR): 6.5 (6.3–6.8).

### HCRU

During follow-up, the IR of ITP-related outpatient visits was 8.9 (95% CI: 8.2–9.5) per person-year, with 7.1 (6.4–7.8) in TPO-RA-naïve and 12.5 (11.2–13.9) in TPO-RA-exposed patients ( Table 5 ). The IR for ITP-related inpatient admissions was 0.1 (0.1–0.2) per person-year, with an IR of 1.1 days (95% CI: 0.8–1.3) per person-year for hospital stays. Overall, patients had an IR of 0.1 (0–0.2) ITP-related accident and emergency department (A&E) visits per person-year. Inpatient and day case admissions, virtual consultations, and A&E visits were similar across the TPO-RA naïve and TPO-RA exposed groups.

**Table 5:** Healthcare resource use in patients with primary ITP while on treatment with avatrombopag.

	Overall, *n* = 76	TPO-RA naïve, *n* = 51	TPO-RA exposed, *n* = 25
Patients with outpatient visits, *n* (%)	58 (76%)	37 (73%)	21 (84%)
All cause, median (IQR)	6.0 (1.0–14.2)	5.0 (0–10.0)	9.0 (2.0–20.0)
ITP-related outpatient visits, IR (95% CI) ^a^	8.9 (8.2–9.5)	7.1 (6.4–7.8)	12.5 (11.2–13.9)
Patients with ITP-related VC, *n* (%)	34 (45%)	19 (37%)	15 (60%)
Median (IQR)	0 (0–5.2)	0 (0–5.0)	1.0 (0–7.0)
ITP-related VC, IR (95% CI) ^a^	3.7 (3.3–4.1)	3.6 (3.1–4.2)	3.8 (3.1–4.6)
Patients with ITP-related A&E visits, *n* (%)	5 (7%)	3 (6%)	2 (8%)
Median (IQR)	0 (0–0)	0 (0–0)	0 (0–0)
ITP-related A&E visits, IR (95% CI) ^a^	0.1 (0–0.2)	0.1 (0–0.2)	0.1 (0–0.3)
Patients with ITP-related day case admissions, *n* (%)	6 (8%)	3 (6%)	3 (12%)
Median (IQR)	0 (0–0)	0 (0–0)	0 (0–0)
ITP-related day case admissions, IR (95% CI) ^a^	0.2 (0.1–0.3)	0.2 (0.1–0.4)	0.1 (0–0.3)
Patients with ITP-related inpatient admissions, *n* (%)	7 (9%)	4 (8%)	3 (12%)
Median (IQR)	0 (0–0)	0 (0–0)	0 (0–0)
ITP-related inpatient admission, IR (95% CI) ^a^	0.1 (0.1–0.2)	0.1 (0–0.2)	0.2 (0–0.4)
Length of hospital stay, days ^b^			
Median (IQR) ^c^	0 (0–0)	0 (0–0)	0 (0–0)
IR (95% CI)	1.1 (0.8–1.3)	0.5 (0.4–0.7)	1.6 (1.2–2.2)

Abbreviation: VC, virtual consultation.

Note: Reported medians represent simple descriptive counts of visits, admissions, or hospital-stay days recorded during the follow-up period. VCs are recorded separately from outpatient visits.

^a^ Incidence rate per person-year.

^b^ The incidence rate for length of hospital stay represents the number of hospital stay days per patient-year. If there were more than one ITP-related inpatient admission, lengths of stay (days) were summed over the entire observation period (total length of stay).

^c^ The majority of patients had day-case admissions, resulting in no overnight stay. Across the cohort, the minimum and maximum lengths of stay ranged from 0 to 38 days overall, 0 to 21 days in the TPO-RA-naïve group, and 0 to 38 days in the TPO-RA-exposed group.

## Discussion

This is the first European multi-country observational study on real-world avatrombopag use in primary ITP, offering a comprehensive view of patient outcomes, characteristics, treatment patterns, and ITP-related HCRU.

The goal of treatment is to maintain a PC of 50 to 150 × 10 ^9^ /L, and by week 12, nearly all patients (93%) achieved a PC threshold of 50 × 10 ^9^ /L. This aligns with the primary effectiveness outcome observed in a prior Phase 3 clinical study (NCT01438840), which found that 66% achieved a PC ≥ 50 × 10 ^9^ /L by day 8. ^13^ Eighty percent of our study population had a complete response (≥100 × 10 ^9^ /L) by week 26, and 87% by week 52 postindex. A recent real-world study in Spain (AVAMAD) including adults with both primary ( *n* = 55) and secondary ( *n* = 11) ITP (15.2% newly diagnosed, 12.1% persistent ITP, 72.7% chronic ITP) reported that 86.4% of the patients were responders (PC of ≥ 50 × 10 ^9^ /L) and 73% had complete response (≥100 × 10 ^9^ /L) after avatrombopag initiation. 20 In a real-world retrospective study of 50 Chinese adults with ITP, 88% were responders (PC ≥ 30 × 10 ^9^ /L) and 70% had a complete response (≥100 × 10 ^9^ /L) after treatment with avatrombopag. 21 A recent U.S.-based observational study including 75 patients with primary or secondary ITP (23 with newly diagnosed/persistent ITP and 52 with chronic ITP) reported that 91% of newly diagnosed/persistent patients versus 96% of chronic patients achieved a platelet response (≥50 × 10 ^9^ /L), and 86% versus 81% achieved a complete response (≥100 × 10 ^9^ /L) on at least one measurement (at least 7 days after initiation of avatrombopag and no more than 6 months after its initiation) without the need for rescue therapy. 22 Similarly, 89% of the chronic patients in our study achieved a complete response, and 96% achieved a platelet response ≥50 × 10 ^9^ /L during follow-up. This was consistent with another U.S.-based study that reported 93% and 86% of patients achieved a PC of ≥50 × 10 ^9^ /L and ≥100 × 10 ^9^ /L, respectively. 17 Data from the Norwegian ITP registry showed that response (PC ≥ 30 × 10 ^9^ /L) to first TPO-RA was achieved by 70% of patients receiving romiplostim, 68% receiving eltrombopag, and 100% receiving avatrombopag. 23 These findings confirm that avatrombopag enabled most patients with ITP to achieve clinically meaningful and durable PC improvements. Although many patients achieved these response thresholds at least once during follow-up, time-specific analyses showed variability in PC over time. This observation indicates that treatment responses may not be consistently maintained across all assessment time points, underscoring the value of longitudinal evaluation when assessing treatment effectiveness.

The median (95% CI) time to achieve a PC ≥ 50 × 10 ^9^ /L in our cohort was 10 (8–14) days from the index date, which was slightly shorter than the reported median of 2 weeks (IQR: 1.3–4.3) in the AVAMAD study, 20 and comparable to the rapid response observed in the Phase 3 clinical trial, in which a majority of patients achieved a PC ≥ 50 × 10 ^9^ /L within the first week. 13

The median baseline PC in our study was 42 × 10 ^9^ /L, which is higher than the baseline PCs reported in the Phase 3 clinical trial (12.5 × 10 ^9^ /L) 13 and the AVAMAD study (36 × 10 ^9^ /L). 20 These differences might be partially due to variations in the timeline and definition of baseline measurement PC across studies (average of three PC prior to avatrombopag in this study) or the possible effect of rescue medications, such as steroids, administered close to the baseline PC measurement. Examining the week prior to the index date, out of 46 patients with a baseline PC > 30 × 10 ^9^ /L in our study, 13 were receiving ongoing steroid treatment at the index date, and three had received IVIg either at the index date or within the preceding week.

Of 26 patients with ongoing steroid treatment at avatrombopag onset, 22 (84.6%) discontinued steroids during the follow-up period, and 3 continued steroid treatment with a reduced dose. A U.S. study found that 57% of patients with ITP receiving concomitant ITP medications before switching to avatrombopag were able to discontinue them after switching, including 63% of those receiving chronic corticosteroids. 17 In another U.S. study based on administrative claims data, 35% of 75 avatrombopag-treated patients receiving concomitant steroids discontinued their use (mean follow-up time of 246 days). 16 Similarly, 33.3% of patients with ITP in the Phase 3 trial 13 and 56% of those in the AVAMAD study 20 reduced or discontinued their medication after initiating avatrombopag. A post hoc analysis of the Phase 3 study (NCT01438840) reported that more than half (57%, 12/21) of patients previously on steroids were able to discontinue (43%) or reduce their dose (14%) after starting avatrombopag. 24 One possible explanation for the favorable results (discontinuing steroids) observed in this study is that many participants were TPO-RA naïve and initiated avatrombopag treatment early in their disease course (a median of 3 years postdiagnosis).

There are limited data on healthcare utilization among patients with primary ITP who use TPO-RA. A U.S. study based on administrative health insurance claims indicated that the hospitalization rate among patients treated with TPO-RAs (avatrombopag, romiplostim, eltrombopag) was 0.6 per person-year, with an average hospital stay of 10 days, 25 which is higher than the rates reported in this study. A Canadian study based on a provincially funded special drug access program also reported an ITP-related hospitalization rate of 0.6 per person-year for patients treated with TPO-RA (romiplostim and eltrombopag). 26 Considering that PC change for the naïve group was higher than that of the exposed group, and that the individuals in the naïve group had lower HCRU, particularly outpatient visits, during the follow-up period, these data suggest that patients with ITP previously exposed to TPO-RA may have a more severe disease profile and, consequently, they may not respond as well to new treatments and require more healthcare.

Rescue therapy was used by 16% of the patients in this study, which is lower than the 21.9% reported in the Phase 3 trial 13 and the 26% observed in the AVAMAD study. 20 A total of 9.7% of patients in a Chinese clinical trial required rescue therapy. 14 It should be noted that 25% of our study participants were diagnosed within 12 months before the index date (newly diagnosed/persistent cases), which falls outside the avatrombopag license for chronic ITP. This may indicate a more pronounced benefit of treatment with avatrombopag in earlier disease stages. However, the sample size was not large enough to evaluate the outcomes for this subgroup.

Avatrombopag treatment was permanently discontinued by 19 (25%) patients in the current study, compared with 14% ( *n* = 6) in the U.S. study. 17 The most common reason for discontinuation in our study was a sustained response and being a candidate for achieving SROT ( *n* = 7, 9%). These individuals achieved CR before discontinuing treatment, and none of them initiated a new treatment during the study period by the end of follow-up (median time from discontinuation to end of study was 366 days). Consistent with previous studies, 17 21 22 23 27 this study provides additional evidence supporting SROT during avatrombopag treatment and suggests that avatrombopag can help maintain response without the need for continuous medication. Five patients (7%) discontinued avatrombopag treatment due to intolerance, and 2 (3%) due to adverse events. The discontinuation reasons in the U.S. switch study included attempted remission (2%), formulary limitations (2%), lack of response (2%), adverse event (headache, PVT; 2%), patient preference (2%), and initiation of rituximab for autoimmune hemolytic anemia (2%). 17 The major reasons for discontinuation in the Phase 3 clinical trial were inadequate therapeutic effect and adverse events, with additional dropouts in the extension phase due to AE (7.7%), patient’s choice (7.7%), inadequate therapeutic effect (5.1%), and being lost to follow-up (2.6%). 13

The proportion of patients with prior corticosteroid exposure in both TPO-RA-naïve and TPO-RA-exposed groups was lower than typically reported in the literature, where rates often exceed 80–90%. 28 29 This may reflect the influence of the COVID-19 pandemic on prescribing behavior, during which guidelines recommended minimizing corticosteroid use and considering TPO-RAs as first-line therapy where appropriate. 30

In this study, nine patients (12%) experienced CTCAE grade ≥ 3 bleeding events, and 3 (4%) had TEs compared with three (6.4%) with grade 3 or 4 bleeding events and four (8.5%) with TEs in the Phase 3 trial ( *n* = 47). 13 The Chinese clinical trial reported no TEs or grade ¾ bleeding events during the 6-week core study phase. 14 A Phase 4 study (NCT04638829) including adults with ITP who switched to avatrombopag from another TPO-RA reported that severe bleeding and TEs were found in 1.7% of the patients. 31 The post hoc analysis of the Phase 3 trial data reported that eight of nine patients with thromboembolism had comorbidities significantly increasing thromboembolic risk, despite normal PC at the time of the TEs. 24 In the current study, TEs occurred in women aged 47–62 years, with PC ranging from 25 × 10 ^9^ /L to 256 × 10 ^9^ /L. No data on comorbidities increasing thromboembolic risk were collected as part of this study. Interim results from the Phase 4 study (NCT04638829; *n* = 200) reported that 10 patients experienced TEs, including atheroembolism, cerebral venous thrombosis, deep vein thrombosis, and pulmonary embolism during follow-up. Of these, eight were from the chronic ITP cohort, and two were from the persistent cohort. 32 The rates were similar in patients aged ≥65 years (6.8%), those with comorbidities (6.7%), and those with prior TPO-RA exposure (6.7–8.0%). 33 The GEPTI study reported that 4.5% (12/268) of patients experienced TEs while on avatrombopag, none of which was fatal. 34 The AVAMAD study reported that only one patient (1.5%) experienced a TE, 20 similar to another U.S. study that found no individuals in the newly diagnosed/persistent group had major bleeding or TEs, while in the chronic group, four patients had bleeding events and one patient experienced a TE (rate of thromboembolic complications in the entire cohort was 1.2 per 100 patient-years of avatrombopag treatment). 22 The rate of TEs in the current study was 4.9 events per 100 patient-years. It should be noted that adverse events were recorded for a shorter duration after avatrombopag initiation in these studies compared with our study. Previous trial studies demonstrate a TE rate per 100 patient-years of 5.5–7.5 for romiplostim 35 36 37 and 4.0 for eltrombopag, 38 as compared with a rate of 0.39–1.5 per 100 person-years in patients with ITP overall (not treated with TPO-RAs) in observational studies. 39 40 41 42 43 Overall, our findings, along with other real-world studies showing a low TE rate, provide further support that avatrombopag is safe and effective in patients with primary ITP.

This study has some limitations. First, potential selection bias may exist, particularly in centers with many TPO-RA-naïve patients, though this was mitigated by enrolling patients consecutively by avatrombopag initiation date. Requiring patient consent may also introduce bias, limiting generalizability. Second, although the 12-week medical record requirement (which was irrespective of treatment duration) allowed inclusion of patients who died early, reducing immortal time bias, those lost to follow-up may differ from those retained. Third, data quality depended on the completeness of retrospective medical records. Fourth, participating centers were selected based on interest and capacity, which may not reflect broader clinical practice. Finally, differences in avatrombopag approval timing and the COVID-19 pandemic may have influenced observation periods. Additionally, as a descriptive study, causal inferences cannot be made.

This study provided detailed insights into the real-world clinical effectiveness and safety of avatrombopag in patients with primary ITP in Europe. The findings align with previous clinical trials and postmarketing evidence, reaffirming the effectiveness and safety of avatrombopag within European clinical practice. The findings show that avatrombopag improves real-world ITP outcomes through timely, sustained PC response, low HCRU and AEs, consistent with clinical trials, regardless of previous TPO-RA response. Moreover, this study provided further real-world evidence of avatrombopag treatment discontinuation due to achieving a maintained response.

**What Is Known On This Topic?**

- Clinical trials show that avatrombopag, a thrombopoietin-receptor agonist (TPO-RA), is a safe and efficacious treatment for patients with primary immune thrombocytopenia (ITP).
- There are limited real-world studies of avatrombopag treatment in Europe, assessing its clinical effectiveness in a diverse patient population with primary ITP.

**What Does This Paper Add?**

- This European multi-center retrospective chart review showed that by week 12, 93% of patients with ITP treated with avatrombopag achieved a platelet count ≥50 × 10 ^9^ /L, aligning with clinical trials. Platelet counts reached ≥50 × 10 ^9^ /L and ≥100 × 10 ^9^ /L in a median of 10 (95% CI: 8–14) and 19 (12–23) days from avatrombopag initiation.
- Thromboembolic rates were 4.9 (95% confidence interval: 1.3–12.5) per 100 patient-years during follow-up.
- The findings show that avatrombopag improves real-world ITP outcomes through timely, sustained platelet count response, low healthcare resource utilization and adverse events, consistent with clinical trials, regardless of previous TPO-RA response.

## Data Availability

The data that support the findings of this study are available upon reasonable request from the corresponding author, Carly Rich (Carly.Rich@sobi.com).

## Supplementary Material

Ergänzendes Material Supplementary Material
