# Supplementary material for: Outcomes, Treatment Patterns, and Resource Use in European Adults with Immune Thrombocytopenia Treated with Avatrombopag
Source: TH Open. 2026 Jul 24;10:a28867426. doi: 10.1055/a-2886-7426 (PMC13494746; doi:10.1055/a-2886-7426)
Supplement: Supplementary file 1 [file 10-1055-a-2886-7426_29129719.pdf]

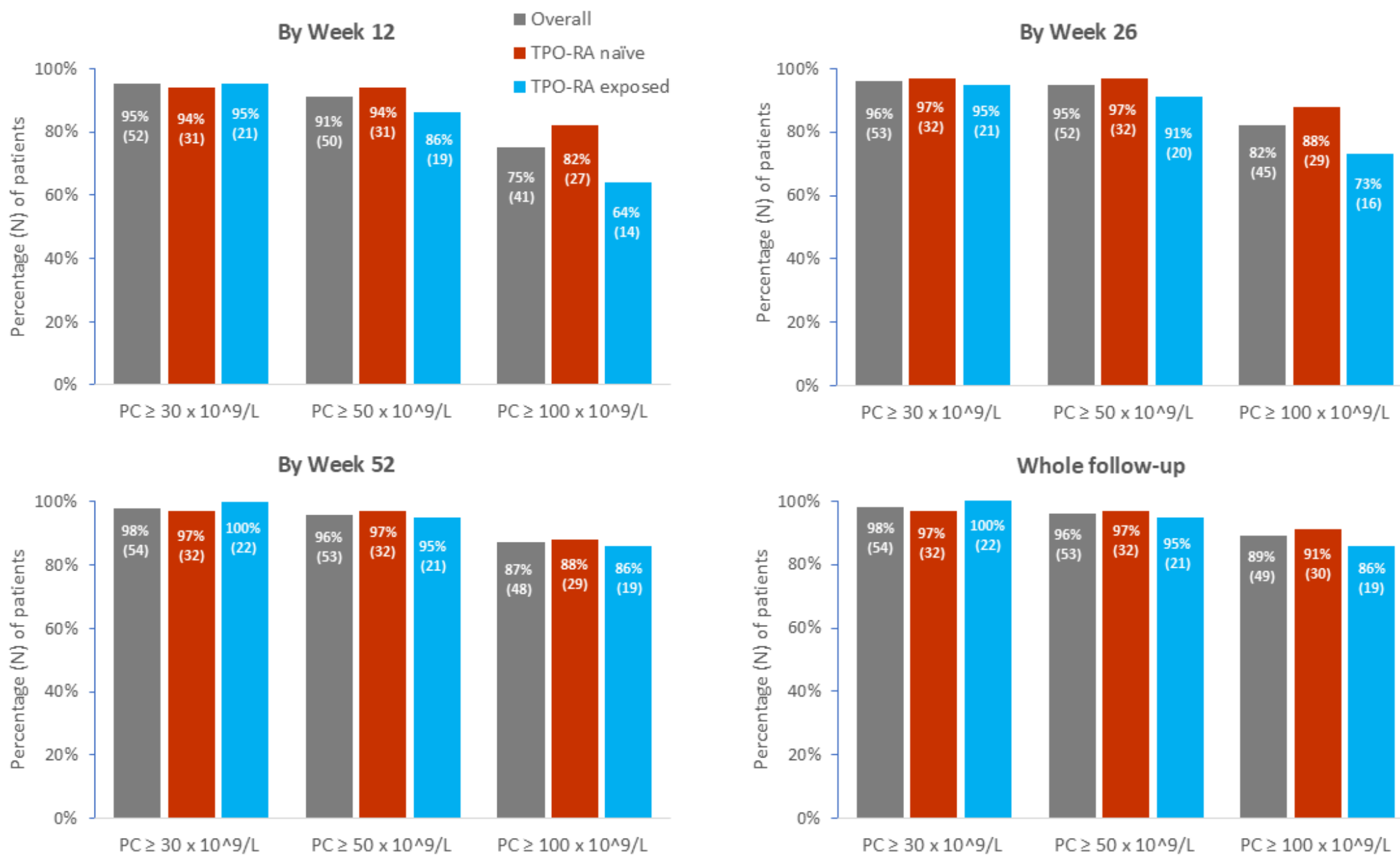

Supplementary Fig. S1. Proportion of patients with chronic ITP (diagnosed >12 months prior to initiation of avatrombopag) achieving meaningful PC thresholds post-index by predefined time points, overall and by prior TPO-RA exposure status.

For analyses describing the proportion of patients who achieved a PC threshold by a particular time point (e.g., by week 12), all measurements between the index date and that time point were included. The total patients with chronic ITP (n = 55) were used as the denominator to provide a conservative estimate of response. Due to variability in follow-up duration, the reported percentages reflect minimum response rates. ITP: immune thrombocytopenia; PC: platelet count; TPO-RA: thrombopoietin-receptor agonist.

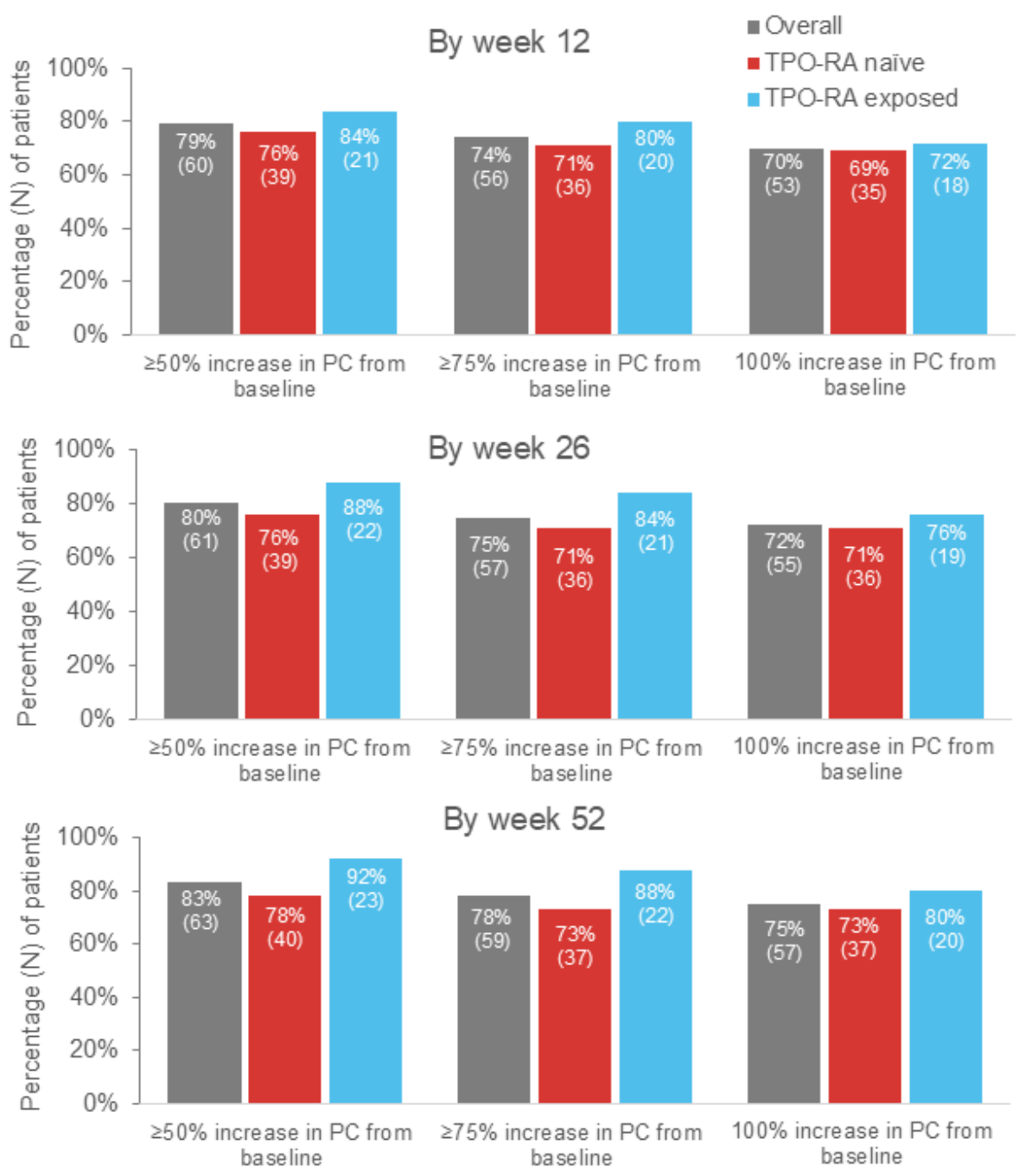

Supplementary Fig. S2. Proportion of patients with primary ITP with a ≥50%, ≥75% and 100% increase in PC from baseline by pre-defined time points, overall and by prior TPO-RA exposure status. For analyses describing the proportion of patients who achieved a PC threshold by a particular time point (e.g., by week 12), all measurements between the index date and that time point were included. PC: platelet count; ITP: immune thrombocytopenia; TPO-RA: thrombopoietin-receptor agonist.

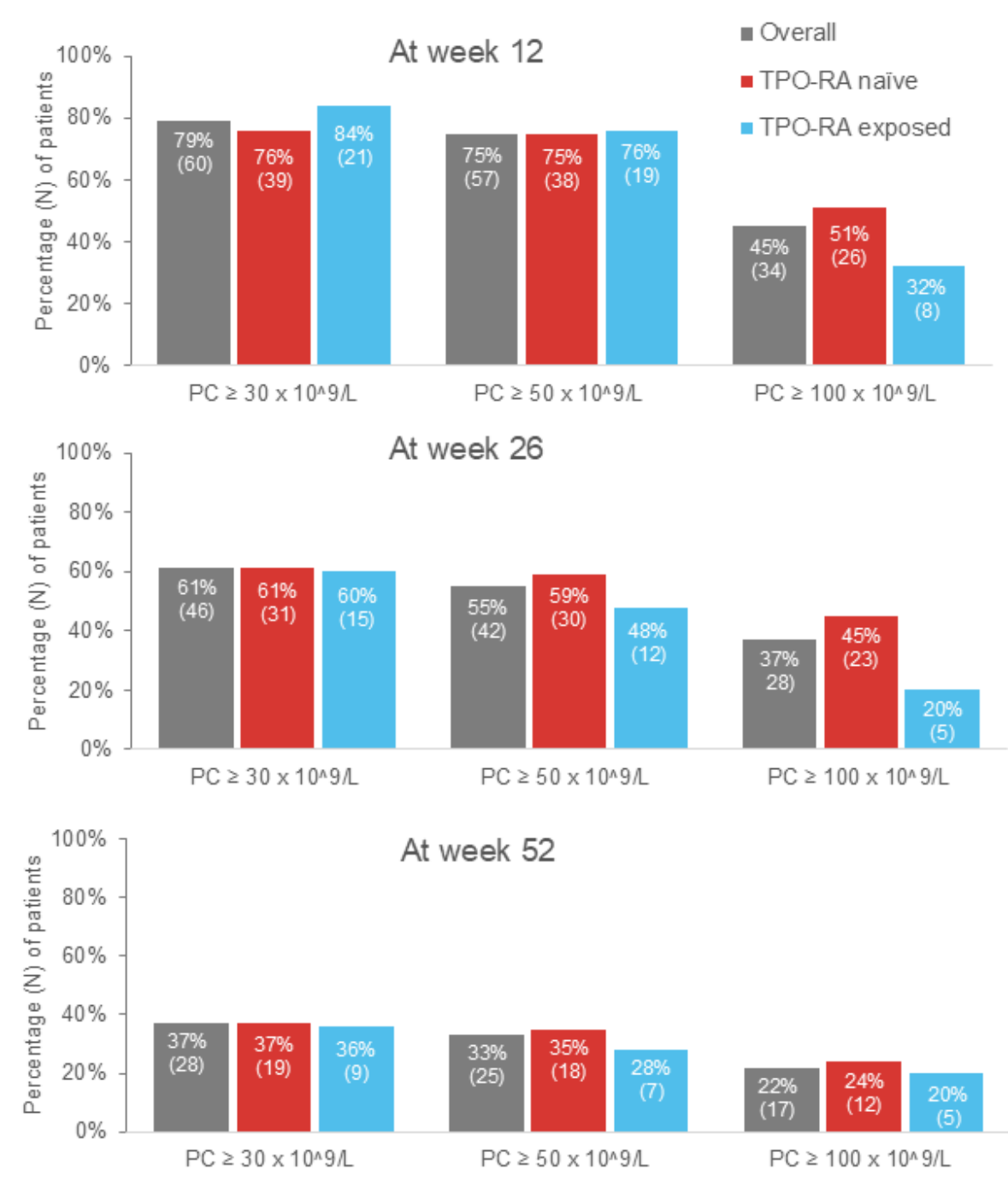

Supplementary Fig. S3. Proportion of patients with primary ITP achieving meaningful PC thresholds post-index at pre-defined time points, overall and by prior TPO-RA exposure status. For analyses considering a PC threshold occurs at a specific time period (e.g., at week 12) an appropriate window was applied around this time point (±4 weeks) to account for variability in appointment scheduling in real world practice. Moreover, only those patients with sufficient follow-up duration were included in these analyses. The reported percentages indicate time-specific snapshots, which more accurately depict the "up-and-down" trajectory of platelet counts during treatment. The total cohort size (n = 76) was used as the denominator to provide a conservative estimate of response. However, variability in follow-up duration and appointment scheduling led to missing data for some patients during the designated follow-up windows. As a result, the reported percentages reflect minimum response rates; PC: platelet count; ITP: immune thrombocytopenia; TPO-RA: thrombopoietin-receptor agonist.

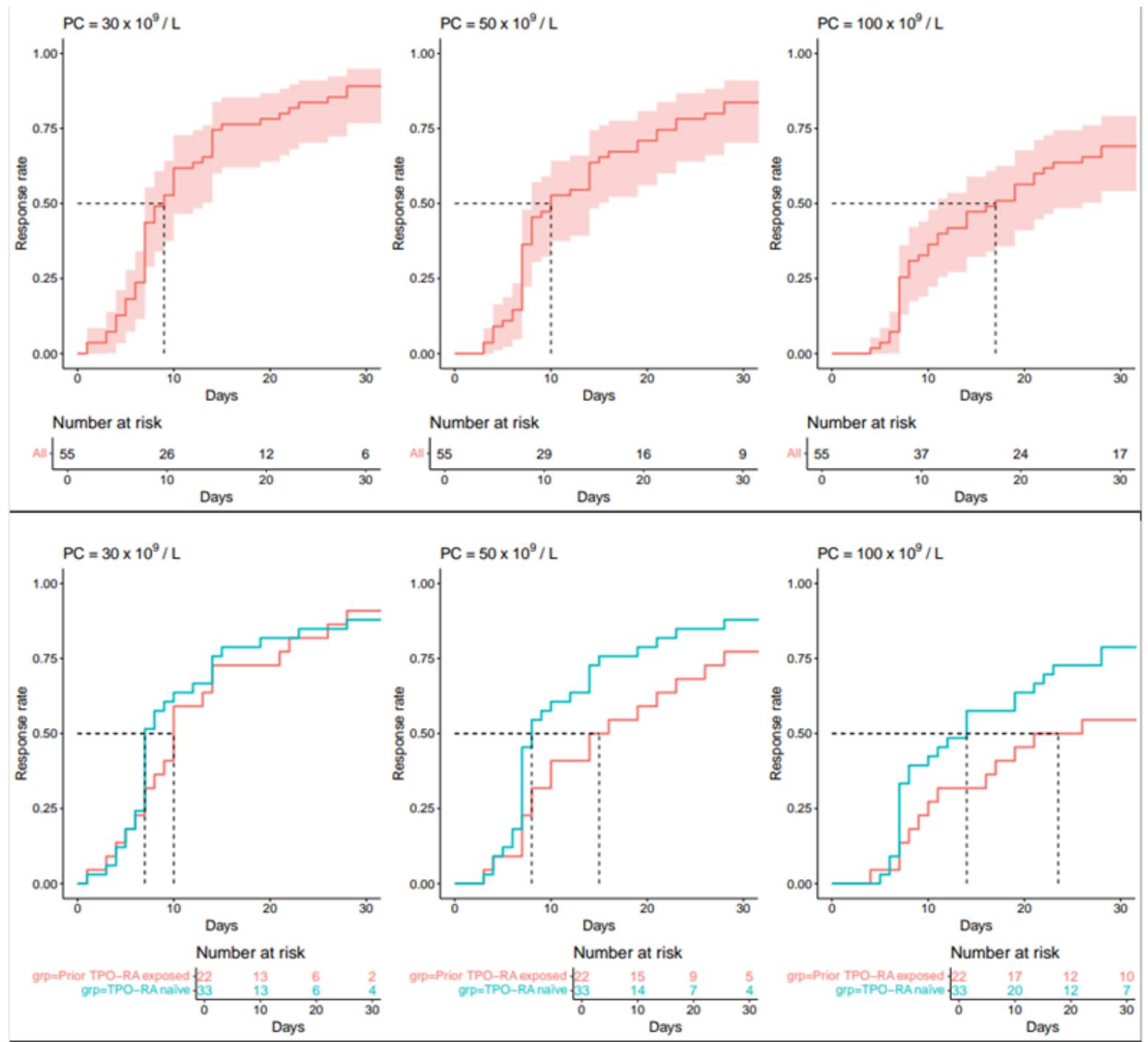

Supplementary Fig. S4. Median time from index date to an initial platelet response, overall (upper row) and by prior TPO-RA exposure status (lower row) among patients with chronic ITP (diagnosed >12 months prior to initiation of avatrombopag). PC: platelet count; TPO-RA: thrombopoietin-receptor agonist; The "number at risk" refers to the total number of patients who are still being observed and have not yet experienced the event of interest (PC response) at the beginning of each time period. This number decreases over time as events occur or as patients are censored. The red shaded area in the top row represents the 95% CIs. The blue line in the lower row indicates the TPO-RA naïve group, while the red line represents the TPO-RA exposed group.

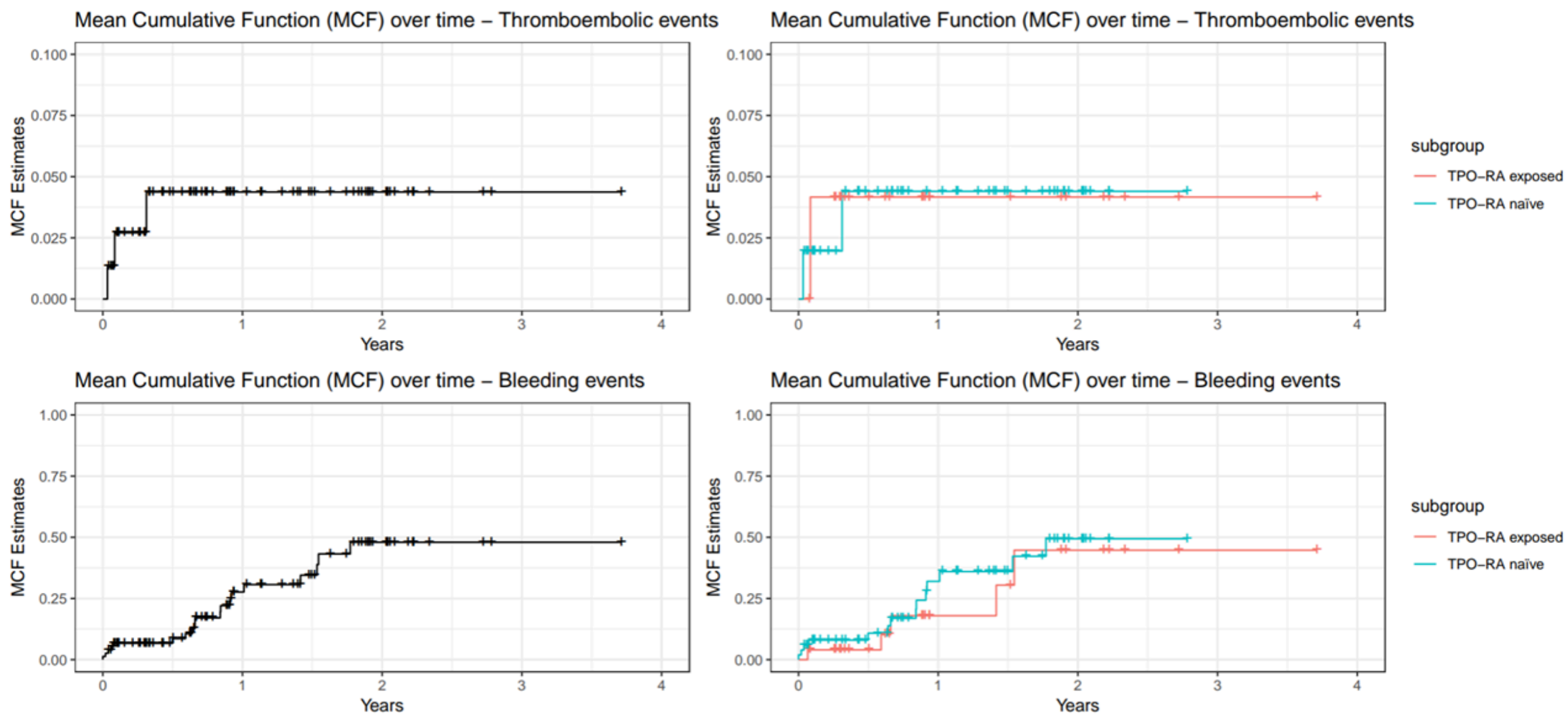

Supplementary Fig. S5. Mean cumulative function (MCF) for thromboembolic and bleeding events, overall and by prior TPO-RA exposure status.

Supplementary Table S1. PC percentage change from baseline at pre-defined time points

	Overall	TPO-RA naïve	TPO-RA exposed
**Overall cohort, N**	76	51	25
Week 12, N	54	33	21
Median (IQR)	160.4% (45.3%-380.4%)	186.5% (91.2%-389.2%)	72.2% (-7.3%-311.1%)
Week 26, N	41	26	15
Median (IQR)	192.9% (34.2%-525.0%)	260.7% (104.9%-506.9%)	51.2% (10.4%-359.4%)
Week 52, N	23	14	9
Median (IQR)	144.2% (37.5%-396.5%)	120.8% (73.5%-389.6%)	144.2% (-47.2%-1757.1%)
**Patients with chronic ITP*, N**	55	33	22
Week 12, N	40	22	18
Median (IQR)	187.0% (53.2%-426.9%)	210.7% (95.8%-400.6%)	88.4% (22.1%-514.1%)
Week 26, N	30	18	12
Median (IQR)	173.2% (29.9%-581.2%)	173.2% (47.4%-534.1%)	122.0% (12.3%-543.8%)
Week 52, N	19	11	8
Median (IQR)	144.2% (14.3%-396.5%)	95.1% (72.3%-318.6%)	319.0% (-47.4%-1767.9%)

*Chronic ITP= diagnosis > 12 months prior to initiation of avatrombopag; IQR: interquartile range; PC: platelet count; TPO-RA: thrombopoietin-receptor agonist.

Supplementary Table S2. Duration of platelet response (sensitivity analyses)

2 weeks gap					
**PC response**	**Maintained response at**		**Overall, N=76**	**TPO-RA naïve, n=51**	**TPO-RA exposed, n=25**
≥30 × 10^9/L	Year 1	Proportion (95% CI)	93.3% (87.9%-99.2%)	94.0% (87.6%-100%)	92.0% (82.0%-100%)
		N patients at time 0	75	50	25
		N at risk at start of year 1	42	30	12
		N events (between year 0 and 1)	5	3	2
	Year 2	Proportion (95% CI)	90.6% (84.3%-97.5%)	94.0% (87.6%-100%)	92.0% (82.0%-100%)
		N patients at time 0	75	50	25
		N at risk at start of year 2	15	8	7
		N events (between year 1 and 2)	0	0	0
≥50 × 10^9/L	Year 1	Proportion (95% CI)	79.6% (70.5%-89.9%)	83.1% (73.1%-94.6%)	71.3% (53.8%-94.5%)
		N patients at time 0	74	50	24
		N at risk at start of year 1	36	27	9
		N events (between year 0 and 1)	14	8	6
	Year 2	Proportion (95% CI)	72.5% (60.3%-87.2%)	83.1% (73.1%-94.6%)	52.8% (31.7%-88.0%)
		N patients at time 0	74	50	24
		N at risk at start of year 2	12	7	5
		N events (between year 1 and 2)	2	0	2
≥100 × 10^9/L	Year 1	Proportion (95% CI)	53.5% (42.0%-68.1%)	59.9% (46.2%-77.6%)	40.6% (23.5%-69.6%)
		N patients at time 0	67	45	22
		N at risk at start of year 1	23	17	6
		N events (between year 0 and 1)	28	16	12
	Year 2	Proportion (95% CI)	49.6% (37.4%-65.8%)	59.9% (46.2%-77.6%)	32.5% (16.1%-65.3%)

		N patients at time 0	67	45	22
		N at risk at start of year 2	5	3	2
		N events (between year 1 and 2)	1	0	1
**4 weeks gap**					
≥30 × 10^9/L	Year 1	Proportion (95% CI)	94.5% (89.4%-99.9%)	96.0% (90.6%-100%)	91.4% (80.7%-100%)
		N patients at time 0	75	50	25
		N at risk at start of year 1	42	30	12
		N events (between year 0 and 1)	4	2	2
	Year 2	Proportion (95% CI)	94.5% (89.4%-99.9%)	96.0% (90.6%-100%)	91.4% (80.7%-100%)
		N patients at time 0	75	50	25
		N at risk at start of year 2	15	8	7
		N events (between year 1 and 2)	0	0	0
≥50 × 10^9/L	Year 1	Proportion (95% CI)	83.8% (75.3%-93.2%)	87.0% (77.8%-97.3%)	76.3% (59.7%-97.5%)
		N patients at time 0	74	50	24
		N at risk at start of year 1	38	28	10
		N events (between year 0 and 1)	11	6	5
	Year 2	Proportion (95% CI)	74.7% (62.6%-89.1%)	87.0% (77.8%-97.3%)	50.9% (29.8%-86.7%)
		N patients at time 0	74	50	24
		N at risk at start of year 2	13	8	5
		N events (between year 1 and 2)	3	0	3
≥100 × 10^9/L	Year 1	Proportion (95% CI)	55.9% (44.3%-70.6%)	59.5% (45.8%-77.4%)	49.1% (30.7%-78.5%)
		N patients at time 0	67	45	22
		N at risk at start of year 1	23	17	6
		N events (between year 0 and 1)	26	16	10
	Year 2	Proportion (95% CI)	55.9% (44.3%-70.6%)	59.5% (45.8%-77.4%)	49.1% (30.7%-78.5%)

		N patients at time 0	67	45	22
		N at risk at start of year 2	6	3	3
		N events (between year 1 and 2)	0	0	0

CI: confidence intervals; TPO-RA: thrombopoietin-receptor agonist; Proportion and 95% confidence intervals were calculated using the Kaplan-Meier estimator, based on the number of events and the number at risk at specific time points.
